# Axillary Hidradenitis

**Published:** 2013-01-18

**Authors:** Sharon S. Stanley, Mark S. Granick

**Affiliations:** Division of Plastic Surgery, New Jersey Medical School—UMDNJ, Newark

**Figure:**
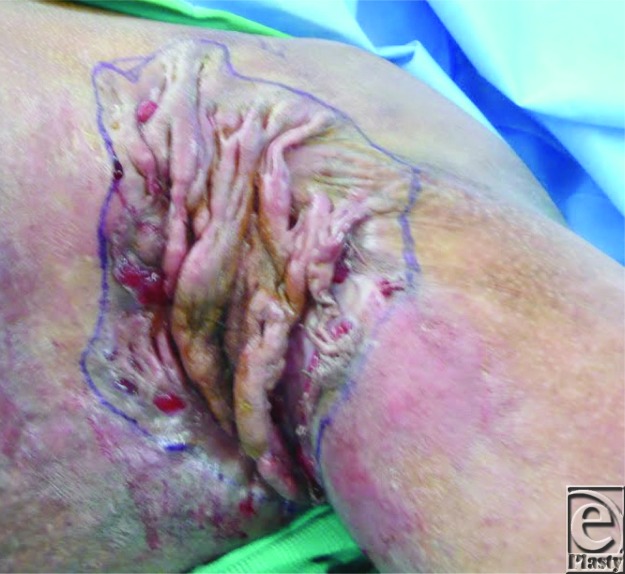


## DESCRIPTION

A 38-year-old black man presented with bilateral end-stage axillary hidradenitis. The photograph of left axillary disease is shown.

## QUESTIONS

1. **Describe the pathophysiology of hidradenitis.**
2. **What are risk factors for hidradenitis?**
3. **What are the treatment options?**

## DISCUSSION

Hidradenitis is a chronic inflammatory skin disease characterized by recurrent nodules and abscesses, typically of apocrine gland-bearing skin. Lesions can progress to sinus tracts and fistulae, and, when healed, can lead to severe scarring and fibrosis. It typically occurs in the groin, inframammary, and axillary regions. It has long been thought of as a disease of the apocrine glands, but more recent literature indicates the etiology to be follicular obstruction. Keratinous plugging of the hair follicle causes dilation of the follicle and eventual rupture. Follicular contents are extruded into the surrounding tissue, which triggers an acute local inflammatory response. Bacteria, if trapped in the follicle, are released as well, and superinfection may be present. However, the role of bacterial infection is controversial as cultures are often sterile, and antibiotics are not always effective. The defect appears to be inherent to the hair follicle, which explains the chronicity and relapsing nature of the disease. Age of onset is typically in the twenties, and the disease tends to wane over the course of 20 years or so with complete cessation in many cases.

Risk factors for hidradenitis are not completely understood but include obesity, smoking, and genetics. Hyperandrogenism had once been thought to play a role, but evidence to support this is conflicting. Obesity is thought to exacerbate the disease process through shearing forces, hormone imbalance, and increased skin surface area. It is also associated with increased levels of circulating cytokines, which contributes to the overall inflammatory process of hidradenitis.

Cigarette smoking appears to play a role although specific causative factors remain to be seen. Nicotine initially stimulates eccrine sweat glands but eventually inhibits glandular function, which may contribute to follicular clogging. It also induces expression of cytokines and stimulates chemotaxis of neutrophils.

Hidradenitis is likely multifactorial, but there is evidence suggesting an autosomal dominant form of the disease. The transmission rate in one study was less than 50%, which would be expected in autosomal dominant inheritance; the authors postulated incomplete penetrance of the gene. Chromosomes 6, 19, and 1 have been associated with hidradenitis, but they cannot account for all cases.

There are currently no established medical therapies to treat active disease and prevent recurrence. Immunosuppressants and antibiotics have limited efficacy. Surgical treatment varies for different stages of the disease. Abscesses should be drained, packed, and left open to avoid closing a possibly contaminated wound. Sinus tracts require unroofing and aggressive exploration of the base as there may be additional tracts. For larger areas of disease, extensive local excision is best. Areas of both active and chronic disease must be removed. Sinus tracts often have visible granulomatous disease that need to be excised. Options for coverage include skin grafting, local transposition flaps, and rarely, pedicled flaps or free tissue transfer. Alternatively, wounds can be left to heal by secondary intention, which often works well.

In this particular patient, Biobrane (UDL Laboratories, Rockford, IL) was used to stabilize the wound following extensive local excision. Biobrane was removed after 2 weeks at which time a split thickness skin graft was placed over the defect. The purpose of Biobrane was to optimize the wound bed for eventual skin grafting. Without it, the graft would be placed on top of the axillary fat pad, a poor recipient for skin grafting. The patient went on to heal uneventfully.

## Figures and Tables

**Figure 1:**
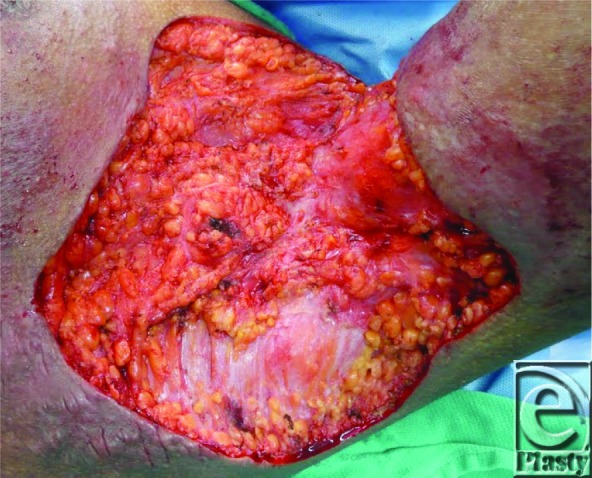
Left axilla following excision.

**Figure 2:**
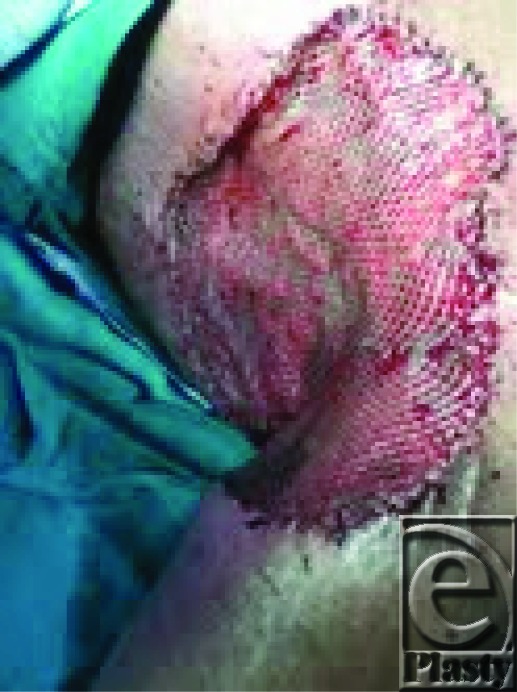
split-thickness skin graft.

**Figure 3:**
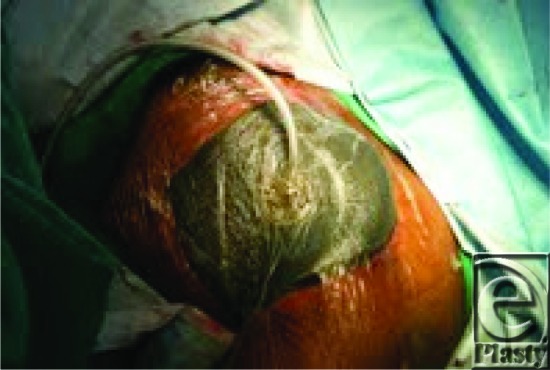
Negative pressure stent dressing.
